# Prognostic factors for conditional survival in patients with muscle-invasive urothelial carcinoma of the bladder treated with radical cystectomy

**DOI:** 10.1038/srep12171

**Published:** 2015-07-27

**Authors:** Minyong Kang, Hyung Suk Kim, Chang Wook Jeong, Choel Kwak, Hyeon Hoe Kim, Ja Hyeon Ku

**Affiliations:** 1 Department of Urology, Seoul National University Bundang Hospital, Seongnam, Kyeonggi-do; 2 Department of Urology, Seoul National University Hospital, Seoul, Republic of Korea

## Abstract

Because only a few studies have evaluated conditional survival (CS) in bladder cancer patients, we examined conditional overall survival (OS) and cancer-specific survival (CSS) in these patients after radical cystectomy (RC), and determined which prognostic variables affect CS over time. We reviewed 487 patients treated with RC and pelvic lymph node dissection at our institution between 1991 and 2012. Cox regression models were used to identify the significant prognostic factors for CS depending on clinicopathological characteristics. As survival time increased after RC, conditional OS and CSS rates increased when compared with baseline survival probability. CS more significantly improved in the patients with unfavorable pathologic characteristics. While many variables were associated with survival at baseline, only age was found to be a significant prognostic factor for 5-year conditional OS in all given survivorships. In conclusion, conditional OS and CSS probabilities significantly improved over time, with greater improvements in the cases with unfavorable pathologic features. Moreover, age remained the key prognostic factor for conditional OS estimates from baseline to 5 years after surgery. Our results provide practical survival information to guide adjustments in our current follow-up strategy for bladder cancer patients after RC.

The accurate prediction of a patient’s prognosis is critical for clinicians to plan the appropriate follow-up strategy after initial cancer treatment. Although the existing tools are helpful for the overall comparison of prognosis, they are less informative for patients who have already survived for a significant period of time after definitive therapy^1^. Conditional survival (CS) is an emerging concept in estimating cancer prognosis and reflects the probability that a patient will continue to survive for additional time, given that the patient has already survived for a period of time after the initial diagnosis or index cancer therapy^2^. CS can more precisely reflect the changes in a patient’s risk over time, allowing clinicians to predict more accurately the prognosis of various cancers^3^,^4^,^5^. CS is more potent in those diseases with unfavorable prognoses, such as lung and colon cancer^6^,^7^. Indeed, patients with long-term survival in these cancers show a better prognosis than those patients immediately after initial treatment.

Bladder cancer, particularly when diagnosed with muscle-invasive disease, also has an unfavorable prognosis, with a 5-year overall and cancer-specific survival estimated of about 60%^8^,^9^. Many patients, including up to 50% of those with infiltrating disease, experience tumor recurrence and progression after curative surgery^10^. Despite the valuable benefits of CS, few studies have evaluated the usefulness of CS in patients with bladder cancer. We examined overall CS after radical cystectomy (RC) with pelvic lymph node dissection (PLND) in bladder cancer patients at a single institution, and determined the significant prognostic variables affecting CS over time.

## Results

**Figure 1:**
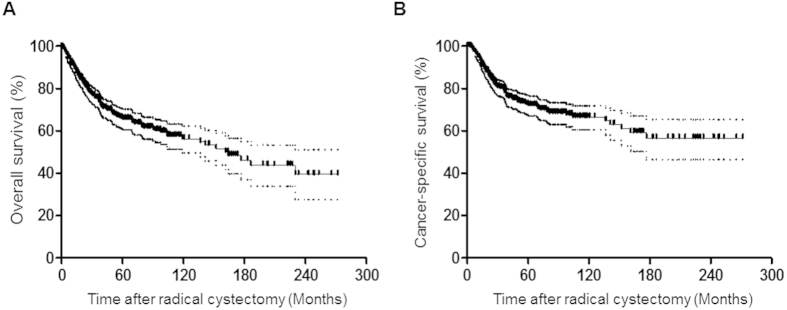
Kaplan-Meier survival curves for median (**A**) overall survival and (**B**) cancer-specific survival of patients with muscle-invasive bladder cancer included in this study. The solid lines represent the Kaplan-Meier estimates, and the dashed lines indicate 95% confidence intervals.

Table 1 presents the baseline characteristics of the patients included in this study. Median age was 63.6 years old and 86.5% of patients were male. Of note, approximately 37% of patients were diagnosed with non-organ confined diseases and about 83% were classified as having a high-grade tumor at the time of surgery. Resection margin positivity was noted in only 1.9% of patients. The number with pathologic lymph node (LN) positivity was 105 (22.2%). After RC, 26.4% of patients were treated with adjuvant systemic chemotherapy. Of 473 patients, all-cause mortality was 34.0% (N = 161) with a median overall survival (OS) of 164.1 months (95% confidence interval [CI] 118.3–209.9; Fig. 1A), and cancer-specific mortality was 25.6% (N = 121) with a median cancer-specific survival (CSS) of 180.86 months (95% CI 165.82–195.89; Fig. 1B).

**Figure 2:**
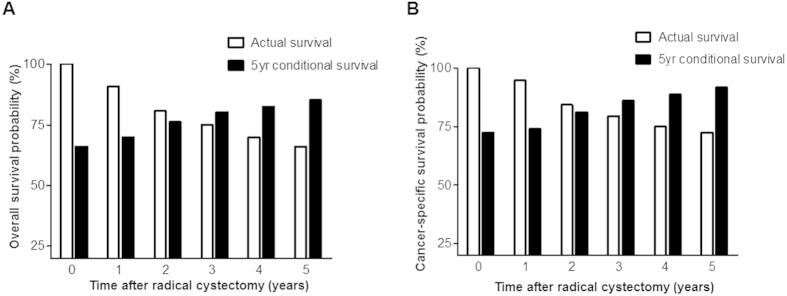
Conditional (**A**) overall and (**B**) cancer-specific 5-year survival rates relative to baseline overall survival rate. The black bar indicates the conditional overall 5-year survival, while the white bar represents the baseline overall 5-year survival of bladder cancer patients who underwent radical cystectomy with pelvic lymph node dissection.

**Figure 3:**
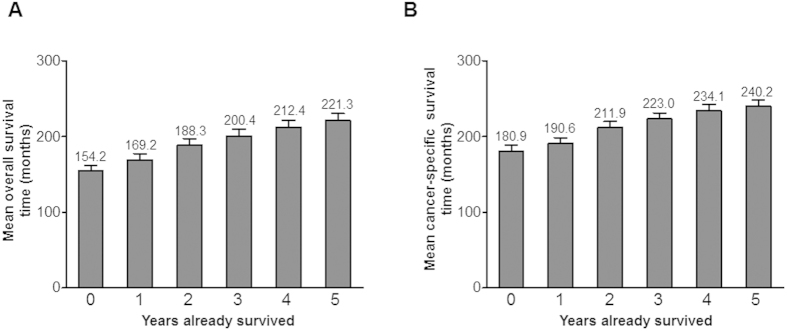
Mean (**A**) overall and (**B**) cancer-specific survival times conditioned on having survived 0 year (473 patients), 1 year (419 patients), 2 years (339 patients), 3 years (260 patients), 4 years (201 patients), and 5 years (162 patients).

We assessed the conditional probability of OS and CSS for additional periods according to years survived after RC (Table S1 and S2, respectively). Notably, as survival time increased after RC, 1-, 2-, 3-, 4-, and 5-year overall and cancer-specific survival estimates also increased compared to the baseline survival probability. For example, 1-, 2-, 3-, 4-, and 5-year conditional OS rates were 88.8%, 82.6%, 76.8%, 72.8%, and 69.9% in patients who survived 1 year after surgery, but were 96.0%, 93.1%, 91.1%, 87.2%, and 85.2% in patients alive 5 years after surgery (Figure S1A). Similarly, 1-, 2-, 3-, 4-, and 5-year conditional CSS estimates were 89.1%, 83.9%, 79.2%, 76.6%, and 74.1% in individuals who survived 1 year after RC, but were 96.6, 94.5, 94.5, 91.7, and 91.7% in patients who survived 5 years after surgery (Figure S1B). As shown in Fig. 2, five-year conditional OS and CSS increased over time from 66.1% and 72.5% at baseline to 85.2% and 91.7% at 5 years after RC, respectively, whereas the actual survival rates notably decreased over time after surgery. Additionally, the mean overall and cancer-specific survival times also increased when conditioned on having survived 0-, 1-, 2-, 3-, 4-, or 5 years after RC (Figs 3A,B, respectively).

We examined whether clinicopathological parameters, including age, gender, and pathologic T stage affected the 5-year conditional OS and CSS rates (Table S3 and S4). In conditional OS estimates, gender and additional treatment with systemic chemotherapy (pre- or post RC) were not significant variables at baseline. Although pathologic T stage, tumor grade, the presence of lymphovascular invasion (LVI), the presence of carcinoma *in situ* (CIS), margin positivity, and LN status obviously affected 5-year conditional OS and CSS at baseline and after a 1-, 2-, or 3-year survivorship, none of these parameters sustained their statistical powers at 4- and 5-year survivorships. Interestingly, age was the only significant factor influencing 5-year conditional OS rates, but not CSS rates, in all given survivorships after RC. Indeed, patients <65 years old had an 18% higher 5-year OS rate than patients >65 years old, and this difference was maintained during all given survival times after RC.

More importantly, we aimed to identify the prognostic factors for the conditional OS and CSS estimates from baseline to 5 years after RC using multivariable cox proportional hazards analysis (Tables 2 and 3, respectively). Although several variables were significantly associated with OS and CSS at baseline, these factors lost their statistical significance over time. Notably, only age remained as a substantial prognostic factor for conditional OS estimates in all given survivorships after cystectomy.

## Discussion

Oncologic outcome is typically expressed by the estimate of survival, based upon the Kaplan-Meier analysis, as to the time from diagnosis (or treatment) to an event of interest^11^. These estimates do not reflect the changes of survival probability over time after initial survival analysis^12^. CS provides real-time information for modified survival estimates, and therefore, it is more helpful for cancer patients to plan their remaining life and clinicians to plan surveillance strategy^13^. Similar to the results with other malignancies, CS rates substantially improved over time after definitive surgery in the patients with muscle-invasive bladder cancer^14^,^15^. Sun *et al.*^14^ reported that the 5-year cancer specific mortality (CSM)-free survival rate was 63.9% at baseline, and improved to 86.3% in patients attaining 5-year survival after surgery. They noted that survival for the initial 2 years after RC was pivotal to the subsequent prognosis of patients. When patients have survived this critical period after RC, their CSM-free survival estimate increased from 64% to >80%. Ploussard *et al.*^15^ also suggested that the risk profile of bladder cancer patients after RC changes over time. The probabilities of 5-year overall CS increased from 60.7% (1 yr survivorship) to 74.3% (10 yr survivorship). Likewise, our data revealed that a longer survivorship after surgery leads to an increase in OS and CSS probability in patients with muscle-invasive bladder cancer. For instance, patients who were alive at 1 year after RC had 70% and 74% 5-year conditional OS and CSS rates, respectively, whereas patients who survived 5 years after surgery had 85% and 92% 5-year conditional OS and CSS rates.

The key observation from our study was that patients with unfavorable pathologic factors had greater improvement in conditional survival in the early time period after RC. Of note, if these patients survived the first 2–3 years after RC, their survival chances drastically increased. Patients with advanced stage tumors (≥pT3) showed markedly improved 5-year conditional OS and CSS rates at 3 years after surgery (45% → 67% in OS; 53% → 83% in CSS). However, their favorable counterparts had comparable 5-year conditional OS and CSS (approximately a 5% increase) at baseline and 3 years after surgery. We observed a similar effect in patients with LVI and lymph node positivity, whose 5-year OS and CSS estimates increased approximately 30–40% between baseline and 3 years after RC, whereas those without LVI or lymph node positivity showed only subtle increases. As reported by Sun *et al.*, CS was considerably influenced in patients with unfavorable clinicopathologic features, including advanced T stage, higher tumor grade, older age, and female gender, more so than in those patients with favorable characteristics^14^. Ploussard and colleagues have also noted that patients with advanced tumor stage showed more remarkable improvements in the 5- and 10-year CS rate^15^. In other malignancies, including brain, breast, and colorectal cancers, CS significantly improves in patients with more advanced stages of cancers^16^,^17^,^18^,^19^. CS merits a role in planning the surveillance strategy for muscle-invasive bladder cancer patients with unfavorable characteristics and a poor prognosis.

Our present study showed clear differences in the 5-year conditional OS and CSS rates between groups having different clinicopathologic features. Notably, contrary to what has been shown in other reports, only age (<65 years vs. ≥65 years) continued to maintain its significance from baseline to 5 year survivorship, particularly in OS estimates. However, well-known prognostic factors such as pathologic T stage, tumor grade, LVI, and margin status lost their significances 2–3 years after surgery. Moreover, in the multivariable Cox proportional hazards model, age was exclusively identified as the significant prognostic factor for the conditional OS estimate, not CSS, when conditioned on all given survivorships. Conversely, the influences of pathologic variables diminished over time with a decreased hazard ratio or statistical power. These results indicate that age independently affects the changing risk over time as well as the probability of being alive for an extended time from surgery.

There are several limitations to our study. First, the results of this study are derived from a retrospective data analysis. Second, we did not include information related to other common prognostic factors, including smoking history, obesity, nutritional status, and the presence of comorbid diseases. Finally, these data are from a single tertiary referral center, and thus, require external validation using a multi-institutional database.

In summary, conditional OS and CSS estimates significantly improve over time in patients with bladder cancer after RC compared to baseline survival probabilities. Particularly, CS more substantially improved in the patients with unfavorable pathologic characteristics whose prognosis was initially poor. Furthermore, well-known prognostic factors, such as pathologic T stage and tumor grade, lost their significances after early follow-up duration, while age remained the key prognostic factor for conditional OS probabilities from baseline to 5 years survivorship after surgery. Our data offer valuable information to guide our current counseling strategy for patients with muscle-invasive bladder cancer after radical surgery.

## Methods

### Ethics statements

The Institutional Review Board (IRB) at Seoul National University Hospital Medical Research Institute approved this study (approval number: H-1410-035-616). Because we retrospectively performed our investigation, the IRB waived the need for informed consent documents from our patients. Patient information was anonymized and de-identified before we carried out the study. All study procedures were carried out in accordance with the Declaration of Helsinki guidelines.

### Study samples

We reviewed the electronic medical records for 487 patients treated with RC and bilateral PLND for bladder cancer at Seoul National University Hospital from January 1991 through December 2012. After excluding 14 patients with non-urothelial carcinoma in the pathologic exam, we finally analyzed 473 patients in this study. There is a partial overlap of patients in this population and a previous study reported by Moon *et al.*^20^.

### Study design

We routinely perform RC with PLND for bladder cancer as reported previously^21^. For pathological examination, surgical specimens were fixed using 10% neutral buffered formalin solution and embedded in a paraffin block. Paraffin-embedded samples were sectioned at 4-mm thickness according to the standard processing protocol. Tissue slides were processed with hematoxylin and eosin staining for histological assessment. Experienced pathologists in our institution reviewed all tissue slides with a standard reporting system. Pathologic T stage was determined by the 2010 American Joint Committee on Cancer staging system. Tumor grade was assigned based on the 2004 World Health Organization/International Society of Urologic Pathology consensus classifications. Lymphovascular invasion (LVI) indicates the presence of tumor emboli within the endothelial space, not covered with muscular layers. A positive surgical margin was defined as the presence of tumor cells within perivesical tissues in surgical specimens. We considered the presence of tumors involving the ureter and urethral margin as negative findings in this study. After surgery, patients were followed up according to our institutional protocol. Patients were evaluated at least every 4 months for the first year, and then every 6 months for the second year. Patients were checked annually after 3 postoperative years. Follow-up examination included a complete physical examination, blood tests, chest radiography, and abdominal-pelvic computed tomography.

### Conditional survival analysis

We used the Kaplan–Meier method to estimate the overall survival and CS. A percent survival and a corresponding two-sided 95% CI were presented. The 95% CI was computed as 1.96 times a standard error in each direction. The method of Greenwood was adopted to calculate the standard error. The statistical definition of CS is previously described as follows: CS (*α*|*β*) = S (*α* + *β*)/S (*α*). CS (*α*|*β*) is the probability of additional survivorship for *α* year, when the patient has already survived for *β* years. S (*t*) is the actual survivorship at time *t*^22^. We defined the overall CS after RC as a primary end point. Overall survival represents the time from RC to death by any causes. We used the Cox proportional hazards regression models to determine significant prognostic factors of CS after RC. We assessed the following variables: age, gender, pathological T stage, tumor grade, LVI, carcinoma *in situ* (CIS), margin status, lymph node (LN) status, resected LN numbers, and neo-adjuvant or adjuvant chemotherapy. When variables were significant in univariable analyses, we further assessed those variables using a multivariable model which was estimated by backward stepwise procedure. For stepwise procedures, the selection criterion was defined as *p* < 0.05. All statistical analyses were conducted using R 2.13.0 (R Development Core Team, Vienna, Austria, http://www.R-project.org) and GraphPad Prism, version 5.01 (GraphPad Software Inc., San Diego, CA, USA).

## Additional Information

**How to cite this article**: Kang, M. *et al.* Prognostic factors for conditional survival in patients with muscle-invasive urothelial carcinoma of the bladder treated with radical cystectomy. *Sci. Rep.* **5**, 12171; doi: 10.1038/srep12171 (2015).

## Supplementary Material

Supplementary Information

## Figures and Tables

**Table 1:** Baseline characteristics.

Variables	
No. of patients	473
Age (year)	63.6 (57.4–69.9)
Gender	
Male	409 (86.5%)
Female	64 (13.5%)
Year of surgery	
1991-2001	47 (10.5%)
2002-2012	399 (89.3%)
Pathologic T stage	
pT0	60 (12.7%)
pTis	39 (8.2%)
pTa	21 (4.4%)
pT1	84 (17.8%)
pT2	94 (19.9%)
pT3	143 (30.2%)
pT4	32 (6.8%)
Tumor grade	
Absence of cancer	60 (12.7%)
Low grade	21 (4.4%)
High grade	392 (82.9%)
Presence of lymphovascular invasion	
Absent	311 (65.8%)
Present	162 (34.2%)
Concomitant carcinoma *in situ* at cystectomy	
Absent	336 (71.0%)
Present	137 (29.0%)
Margin status	
Negative	464 (98.1%)
Positive	9 (1.9%)
Pathologic N stage	
pN0	368 (77.8%)
pN1	41 (8.7%)
pN2	54 (11.4%)
pN3	10 (2.1%)
No. of lymph node removed	13 (8-20)
Neoadjuvant chemotherapy	
Not done	423 (89.4%)
Done	50 (10.6%)
Adjuvant chemotherapy	
Not done	348 (73.6%)
Done	125 (26.4%)
Number of deaths	
All-cause mortality	161 (34.0%)
Disease-specific mortality	121 (25.6%)

Data presented are median (interquartile range) or number (%).

**Table 2:** Multivariable Cox proportional hazards analysis for identifying the prognostic factors of five-year conditional overall survival from baseline to 5 years after radical cystectomy.

	†Hazard ratio (95% CI) conditional on survivorship					
Variables	Baseline	1 yr	2 yr	3 yr	4 yr	5 yr
Age	1.04 (1.02–1.07)	1.03 (1.01–1.05)	1.05 (1.02–1.07)	1.06 (1.02–1.10)	1.07 (1.02–1.12)	1.07 (1.01–1.12)
Year of surgery						
1991–2001			Reference	Reference		
2002–2012			1.63 (0.58–4.57)	2.06 (0.64–6.62)		
pT stage						
≤pT2	Reference	Reference	Reference			
≥pT3	2.68 (1.84–3.90)	2.28 (1.52–3.43)	1.26 (0.68–2.29)			
Tumor grade						
Low	Reference					
High	0.82 (0.48–1.41)					
LVI						
Negative	Reference	Reference	Reference			
Positive	1.53 (1.05–2.22)	1.77 (1.17–2.67)	1.83 (1.07–3.12)			
CIS						
Negative	Reference					
Positive	0.83 (0.54–1.26)					
Margin status						
Negative	Reference	Reference	Reference			
Positive	1.12 (0.48–2.61)	1.16 (0.41–3.26)	2.41 (0.53–10.95)			
LN status						
Negative	Reference	Reference	Reference	Reference		
Positive	2.31 (1.62–3.30)	2.42 (1.60–3.66)	2.29 (1.28–4.11)	2.45 (1.19–5.03)		
No. of LN removed						
≥20	Reference	Reference	Reference			
≤19	2.11 (1.33–3.33)	2.30 (1.30–4.06)	3.05 (1.09–8.52)			

^†^Only statistically significant variables in univariable analysis enter into multivariable model. Blank cells represent the non-significant variables in univariable analysis in given survivorship.

LVI: lymphovascular invasion, CIS: carcinoma *in situ,* LN: lymph node.

**Table 3:** Multivariable Cox proportional hazards analysis for identifying the prognostic factors of five-year conditional cancer-specific survival from baseline to 5 years after radical cystectomy.

	†Hazard ratio (95% CI) conditional on survivorship					
Variables	Baseline	1 yr	2 yr	3 yr	4 yr	5 yr
Age	1.02 (1.00–1.04)	1.01 (0.99–1.04)				
pT stage						
≤pT2	Reference	Reference				
≥pT3	2.56 (1.69–3.88)	2.32 (1.48–3.66)				
Tumor grade						
Low	Reference	Reference				
High	1.12 (0.56–2.28)	1.06 (0.49–2.25)				
LVI						
Negative	Reference	Reference	Reference			
Positive	1.75 (1.14–2.68)	1.83 (1.14–2.96)	1.78 (1.01–3.15)			
Margin status						
Negative	Reference	Reference	Reference			
Positive	0.99 (0.39–2.52)	1.21 (0.43–3.42)	2.98 (0.67–13.36)			
LN status						
Negative	Reference	Reference	Reference	Reference		
Positive	2.89 (1.95–4.28)	2.75 (1.77–4.27)	2.54 (1.37–4.72)	2.39 (1.08–5.28)		
No. of LN removed						
≥20	Reference					
≤19	2.18 (1.29–3.67)					

^†^Only statistically significant variables in univariable analysis enter into multivariable model. Blank cells represent the non-significant variables in univariable analysis in given survivorship.

LVI: lymphovascular invasion, LN: lymph node.
