# Supplementary material for: Prognostic factors for conditional survival in patients with muscle-invasive urothelial carcinoma of the bladder treated with radical cystectomy
Source: Sci Rep. 2015 Jul 27;5:12171. doi: 10.1038/srep12171 (PMC4515743; doi:10.1038/srep12171)

Prognostic factors for conditional survival in patients with muscle-invasive urothelial carcinoma of the bladder treated with radical cystectomy

Minyong Kang[1], Hyung Suk Kim[2], Chang Wook Jeong[2], Choel Kwak[2], Hyeon Hoe Kim[2] and Ja Hyeon Ku[2,*]

[1]*Department of Urology, Seoul National University Bundang Hospital, Seongnam, Kyeonggi-do;* [2]*Department of Urology, Seoul National University Hospital, Seoul, Republic of Korea*

Table S1. Conditional overall survival for additional periods by time since radical cystectomy

Years survived after RC (no. at risk)	Conditional overall survival (95% CI) of given year				
	1 yr	2 yr	3 yr	4 yr	5 yr
0 yr (473)	90.8% (89.3-93.3)	80.7% (77.0-84.4)	75.0% (70.9-79.1)	69.8% (65.3-74.3)	66.1% (61.2-71.0)
1 yr (419)	88.8% (85.7-91.9)	82.6% (78.9-86.3)	76.8% (72.3-81.3)	72.8% (67.9-77.7)	69.9% (64.8-75.0)
2 yr (339)	92.9% (90.0-95.8)	86.5% (82.2-90.8)	81.9% (77.2-86.6)	78.6% (73.5-83.7)	76.2% (70.7-81.7)
3 yr (260)	93.1% (90.0-96.2)	88.1% (83.8-92.4)	84.6% (79.7-89.8)	82.0% (76.7-87.3)	80.3% (74.7-85.9)
4 yr (201)	94.7% (91.7-97.8)	90.9% (86.6-95.2)	88.1% (83.2-93.0)	86.3% (80.8-91.8)	82.5% (75.8-89.2)
5 yr (162)	96.0% (92.9-99.1)	93.1% (90.0-97.2)	91.1% (86.2-96.0)	87.2% (80.7-93.7)	85.2% (77.8-92.6)

RC: radical cystectomy

Table S2. Conditional cancer-specific survival for additional periods by time since radical cystectomy

Years survived after RC (no. at risk)	Conditional cancer-specific survival (95% CI) of given year				
	1 yr	2 yr	3 yr	4 yr	5 yr
0 yr (473)	94.7% (93.0-97.2)	84.3% (81.2-88.0)	79.5% (75.9-83.7)	75.0% (70.9-79.6)	72.5% (68.2-77.4)
1 yr (419)	89.1 (86.4-92.6)	83.9 (80.6-87.9)	79.2 (75.3-83.8)	76.6 (72.4-81.6)	74.1 (69.5-79.3)
2 yr (339)	94.3 (92.2-97.5)	88.9 (85.8-93.2)	86.0 (82.3-90.8)	83.2 (79.0-88.6)	81.2 (76.7-86.9)
3 yr (260)	94.3 (92.0-97.9)	91.3 (88.3-95.8)	88.9 (85.3-93.7)	86.2 (82.0-91.9)	86.2 (82.0-91.9)
4 yr (201)	96.8 (93.0-98.6)	93.5 (88.6-96.3)	91.4 (85.8-94.8)	91.4 (85.8-94.8)	88.8 (81.8-93.2)
5 yr (162)	96.6 (92.1-98.5)	94.5 (89.2-97.2)	94.5 (89.2-97.2)	91.7 (84.6-95.5)	91.7 (84.6-95.5)

RC: radical cystectomy

Table S3. Univariable analysis for comparison of five-year conditional overall survival rates according to clinicopathological characteristics

	Years survived after RC					
Variables	Baseline	1 yr	2 yr	3 yr	4 yr	5 yr
Cohort, no.	473	419	339	260	201	162
Age						
<65	74.1 ± 0.3	75.8 ± 3.1	79.8 ± 3.3	83.3 ± 3.3	88.1 ± 3.3	91.0 ± 3.1
≥65	55.6 ± 0.4	60.5 ± 4.7	69.7 ± 5.2	73.9 ± 5.9	66.1 ± 9.8	72.8 ± 10.4
P value	<0.001	<0.001	0.009	0.021	0.002	0.012
Gender						
Male	65.0 ± 2.7	68.8 ± 2.9	75.0 ± 3.1	78.8 ± 3.5	80.7 ± 3.9	86.1 ± 3.8
Female	73.4 ± 0.6	77.6 ± 6.8	84.3 ± 6.5	89.2 ± 5.9	92.1 ± 5.3	92.1 ± 5.3
P value	0.535	0.182	0.268	0.295	0.481	0.970
Year of surgery						
1991-2001	72.3 ± 6.5	76.2 ± 6.6	88.9 ± 5.2	91.4 ± 4.7	91.1 ± 4.9	91.1 ± 4.9
2002-2012	65.9 ± 2.8	70.7 ± 3.0	74.6 ± 3.3	79.0 ± 3.5	79.2 ± 5.3	85.2 ± 5.4
P value	0.328	0.171	0.021	0.036	0.064	0.261
pT stage						
≤pT2	77.8 ± 2.8	80.6 ± 2.8	79.7 ± 3.1	83.0 ± 3.2	85.6 ± 3.7	90.2 ± 3.4
≥pT3	45.4 ± 4.1	48.4 ± 4.9	67.3 ± 5.8	76.4 ± 5.8	74.3 ± 7.8	78.8 ± 7.8
P value	<0.001	<0.001	0.030	0.215	0.210	0.147
Tumor grade						
Low	84.7 ± 4.6	86.6 ± 4.8	81.7 ± 6.1	77.6 ± 7.0	78.3 ± 8.4	80.1 ± 8.3
High	62.6 ± 2.8	66.6 ± 3.0	75.4 ± 3.2	81.4 ± 3.2	83.7 ± 3.7	89.2 ± 3.4
P value	0.016	0.051	0.787	0.223	0.194	0.030
LVI						
Negative	76.5 ± 2.7	79.9 ± 2.8	80.5 ± 3.1	81.7 ± 3.4	84.6 ± 3.9	87.1 ± 3.8
Positive	46.0 ± 4.4	49.3 ± 5.0	65.4 ± 5.8	76.3 ± 5.8	76.8 ± 7.0	87.0 ± 6.6
P value	<0.001	<0.001	0.007	0.223	0.113	0.733
CIS						
Negative	63.5 ± 2.9	68.0 ± 3.1	76.7 ± 3.2	81.4 ± 3.3	82.5 ± 3.9	86.7 ± 3.8
Positive	72.3 ± 4.9	74.4 ± 5.3	73.9 ± 6.4	77.1 ± 6.4	83.5 ± 6.3	89.8 ± 5.6
P value	0.033	0.125	0.676	0.777	0.866	0.889
Margin status						
Negative	66.6 ± 2.5	70.2 ± 2.7	76.3 ± 2.9	80.8 ± 2.9	83.0 ± 3.4	87.8 ± 3.2
Positive	16.9 ± 15.0	22.2 ± 19.2	33.3 ± 27.2	-	-	-
P value	<0.001	0.001	0.003	-	-	-

LN status						
Negative	73.9 ± 2.6	76.2 ± 2.8	79.0 ± 2.9	81.6 ± 3.1	83.9 ± 3.4	88.1 ± 3.2
Positive	37.6 ± 5.5	43.1 ± 6.6	60.6 ± 8.3	72.0 ± 8.8	67.7 ± 16.5	74.7 ± 17.5
P value	<0.001	<0.001	<0.001	0.030	0.239	0.408
No. of LN removed						
≥20	79.4 ± 4.8	84.7 ± 4.8	92.6 ± 4.4	93.9 ± 4.3	96.2 ± 3.8	100.0 ± 0.0
≤19	62.7 ± 2.8	66.7 ± 3.0	73.2 ± 3.2	78.2 ± 3.3	80.5 ± 3.9	85.2 3.7
P value	0.004	0.010	0.010	0.087	0.223	0.251
NACH						
Not done	65.5 ± 2.6	69.5 ± 2.8	76.6 ± 3.0	80.4 ± 3.1	82.8 ± 3.6	87.5 ± 3.4
Done	70.6 ± 7.4	72.9 ± 8.0	73.2 ± 9.1	80.5 ± 9.0	83.6 ± 8.8	88.9 ± 8.1
P value	0.510	0.552	0.902	0.610	0.889	0.901
ACH						
Not done	64.1 ± 2.9	78.1 ± 3.2	75.3 ± 3.4	79.9 ± 3.5	85.3 ± 3.7	89.5 ± 3.5
Done	71.3 ± 4.5	72.6 ± 5.0	78.3 ± 0.5	81.1 ± 5.4	76.1 ± 7.3	81.5 ± 7.3
P value	0.200	0.229	0.713	0.766	0.593	0.754

†Percent survival estimates are shown with standard error calculated by Greenwood method.

LVI: lymphovascular invasion, CIS: carcinoma *in situ*, LN: lymph node, NACH: neoadjuvant chemotherapy, ACH: adjuvant chemotherapy.

Table S4. Univariable analysis for comparison of five-year conditional cancer-specific survival rates according to clinicopathological characteristics

	Years survived after RC					
Variables	Baseline	1 yr	2 yr	3 yr	4 yr	5 yr
Cohort, no.	473	419	339	260	201	162
Age						
<65	78.0 ± 2.9	78.2 ± 3.0	82.4 ± 3.2	86.3 ± 3.1	89.4 ± 3.1	92.4 ± 2.8
≥65	65.2 ± 3.9	67.7 ± 4.4	79.6 ± 4.4	86.2 ± 4.2	84.1 ± 8.7	86.9 ± 8.7
P value	0.001	0.013	0.191	0.435	0.599	0.490
Gender						
Male	71.7 ± 2.6	73.1 ± 2.7	80.1 ± 2.9	85.0 ± 2.8	87.4 ± 3.3	90.8 ± 3.1
Female	78.5 ± 5.9	80.9 ± 6.2	88.0 ± 5.7	93.1 ± 4.8	96.2 ± 3.8	96.2 ± 3.8
P value	0.418	0.251	0.360	0.305	0.412	0.724
Year of surgery						
1991-2001	74.0 ± 6.5	76.2 ± 6.6	88.9 ± 5.8	91.4 ± 4.7	91.1 ± 4.9	91.1 ± 4.9
2002-2012	73.5 ± 2.7	76.2 ± 2.7	81.1 ± 3.0	86.0 ± 2.9	88.1 ± 4.3	92.1 ± 4.1
P value	0.925	0.820	0.156	0.218	0.326	0.754
pT stage						
≤pT2	82.7 ± 2.6	83.6 ± 2.7	83.6 ± 2.9	87.4 ± 2.8	90.9 ± 2.9	94.4 ± 2.5
≥pT3	53.6 ± 4.3	54.4 ± 5.0	75.0 ± 5.5	82.8 ± 5.3	82.4 ± 7.3	84.2 ± 7.3
P value	<0.001	<0.001	0.097	0.432	0.430	0.172
Tumor grade						
Low	91.6 ± 3.7	90.1 ± 4.4	88.2 ± 5.1	88.2 ± 5.1	91.5 ± 4.7	93.7 ± 4.3
High	69.0 ± 2.7	71.0 ± 2.9	80.1 ± 3.0	86.1 ± 2.8	88.0 ± 3.4	91.2 ± 3.2
P value	0.002	0.009	0.327	0.772	0.399	0.227
LVI						
Negative	83.1 ± 2.4	84.2 ± 2.5	85.7 ± 2.8	88.8 ± 2.7	92.2 ± 2.8	93.5 ± 2.7
Positive	52.0 ± 4.6	53.2 ± 5.1	70.0 ± 5.7	79.4 ± 5.6	79.9 ± 6.9	87.0 ± 6.6
P value	<0.001	<0.001	0.003	0.106	0.068	0.458
CIS						
Negative	69.7 ± 2.8	72.3 ± 3.0	82.4 ± 2.9	87.9 ± 2.6	89.8 ± 3.1	92.4 ± 3.0
Positive	79.3 ± 4.6	78.1 ± 5.1	76.9 ± 6.2	80.3 ± 6.3	85.5 ± 6.1	89.8 ± 5.6
P value	0.028	0.192	0.805	0.414	0.327	0.428
Margin status						
Negative	73.1 ± 2.4	74.5 ± 2.5	81.5 ± 2.6	85.9 ± 2.5	88.5 ± 2.9	91.6 ± 2.7
Positive	19.0 ± 16.8	22.2 ± 19.2	33.3 ± 27.2	-	-	-
P value	0.001	0.001	0.001			
LN status						
Negative	81.6 ± 2.3	80.9 ± 2.6	84.3 ± 2.7	87.7 ± 2.6	88.7 ± 3.0	91.0 ± 2.9
Positive	39.6 ± 5.7	46.2 ± 6.4	64.9 ± 7.6	77.1 ± 7.7	90.7 ± 6.3	90.7 ± 6.3
P value	<0.001	<0.001	<0.001	0.027	0.825	0.765
No. of LN removed						
≥20	82.6 ± 4.8	71.6 ± 2.9	79.0 ± 2.9	85.0 ± 2.8	87.7 ± 3.2	90.5 ± 3.0
≤19	69.7 ± 2.7	84.7 ± 4.8	92.6 ± 4.4	93.9 ± 4.3	96.2 ± 3.8	96.2 ± 3.8
P value	0.017	0.067	0.080	0.414	0.825	0.751
NACH						
Not done	71.9 ± 2.5	73.5 ± 2.7	80.8 ± 2.8	85.7 ± 2.7	88. 3 ± 3.0	91.7 ± 2.8
Done	78.5 ± 7.3	79.4 ± 7.2	85.9 ± 6.7	91.2 ± 6.0	94.7 ± 5.1	94.7 ± 5.1
P value	0.467	0.335	0.540	0.395	0.455	0.745

ACH						
Not done	70.6 ± 0.3	71.6 ± 3.1	79.7 ± 3.2	85.1 ± 3.1	89.1 ± 3.3	91.9 ± 3.1
Done	77.4 ± 4.2	80.4 ± 4.3	84.7 ± 4.4	88.6 ± 4.2	88.0 ± 5.5	91.2 ± 5.2
P value	0.179	0.102	0.356	0.507	0.861	0.816

[†]Percent survival estimates are shown with standard error calculated by Greenwood method. LVI: lymphovascular invasion, CIS: carcinoma *in situ*, LN: lymph node, NACH: neoadjuvant chemotherapy, ACH: adjuvant chemotherapy.

Figure S1. Comparison of conditional probabilities of (A) overall survival (1 to 5 years) and (B) cancer-specific survival (1 to 5 years) between two groups who had different survivorship (1yr versus 5yr) after radical cystectomy.

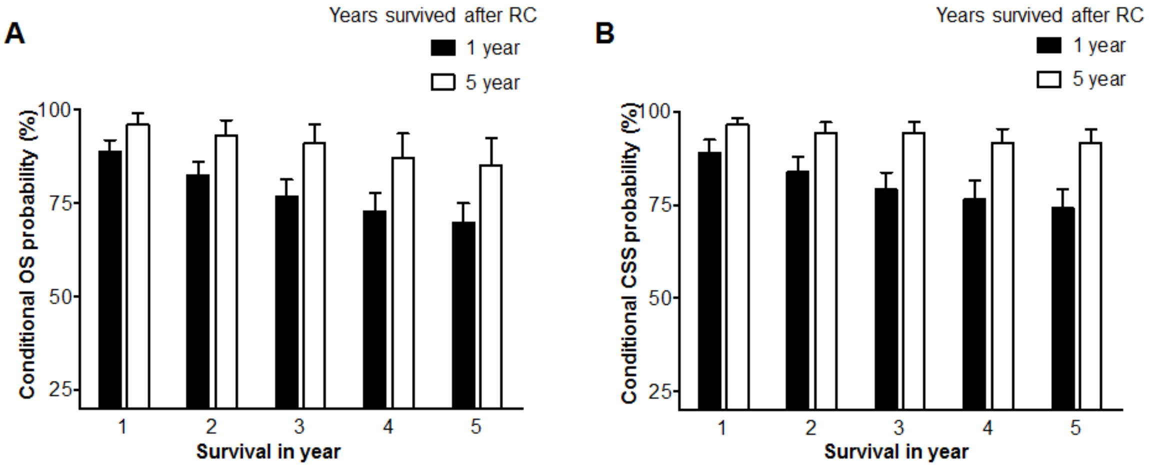

Supplement: Supplementary Information [file srep12171-s1.pdf]
